# A tunable l-arabinose-inducible expression plasmid for the acetic acid bacterium *Gluconobacter oxydans*

**DOI:** 10.1007/s00253-020-10905-4

**Published:** 2020-09-25

**Authors:** Philipp Moritz Fricke, Tobias Link, Jochem Gätgens, Christiane Sonntag, Maike Otto, Michael Bott, Tino Polen

**Affiliations:** IBG-1: Biotechnology, Institute of Bio- and Geosciences, Forschungszentrum Jülich GmbH, 52425 Jülich, Germany

**Keywords:** AraC, P_*BAD*_ promoter, Induction, mNeonGreen, β-d-Glucuronidase UidA, Membrane-bound dehydrogenase

## Abstract

**Abstract:**

The acetic acid bacterium (AAB) *Gluconobacter oxydans* incompletely oxidizes a wide variety of carbohydrates and is therefore used industrially for oxidative biotransformations. For *G. oxydans*, no system was available that allows regulatable plasmid-based expression. We found that the l-arabinose-inducible P_*BAD*_ promoter and the transcriptional regulator AraC from *Escherichia coli* MC4100 performed very well in *G. oxydans*. The respective pBBR1-based plasmids showed very low basal expression of the reporters β-glucuronidase and mNeonGreen, up to 480-fold induction with 1% l-arabinose, and tunability from 0.1 to 1% l-arabinose. In *G. oxydans* 621H, l-arabinose was oxidized by the membrane-bound glucose dehydrogenase, which is absent in the multi-deletion strain BP.6. Nevertheless, AraC-P_*BAD*_ performed similar in both strains in the exponential phase, indicating that a gene knockout is not required for application of AraC-P_*BAD*_ in wild-type *G. oxydans* strains. However, the oxidation product arabinonic acid strongly contributed to the acidification of the growth medium in 621H cultures during the stationary phase, which resulted in drastically decreased reporter activities in 621H (pH 3.3) but not in BP.6 cultures (pH 4.4). These activities could be strongly increased quickly solely by incubating stationary cells in d-mannitol-free medium adjusted to pH 6, indicating that the reporters were hardly degraded yet rather became inactive. In a pH-controlled bioreactor, these reporter activities remained high in the stationary phase (pH 6). Finally, we created a multiple cloning vector with *araC*-P_*BAD*_ based on pBBR1MCS-5. Together, we demonstrated superior functionality and good tunability of an AraC-P_*BAD*_ system in *G. oxydans* that could possibly also be used in other AAB.

**Key points:**

• *We found the AraC-P*_*BAD*_ *system from E. coli MC4100 was well tunable in G. oxydans.*

•  *In the absence of AraC or* *l-arabinose, expression from P*_*BAD*_ *was extremely low.*

•* This araC-P*_*BAD*_ *system could also be fully functional in other acetic acid bacteria.*

**Electronic supplementary material:**

The online version of this article (10.1007/s00253-020-10905-4) contains supplementary material, which is available to authorized users.

## Introduction

Controlled expression of target genes to produce proteins for various purposes in a bacterial cell culture at a defined time point is often beneficial or even required in basic research and in biotechnological applications. For inducible expression, various plasmids have been developed and established in many bacteria including *Escherichia coli*, *Pseudomonas* sp., *Ralstonia* sp., *Bacillus* sp., *Lactoccocus* sp., *Streptomyces* sp., mycobacteria, corynebacteria, halophilic bacteria, and others (reviewed in, for example, Chen 2012; Connell 2001; Dilworth et al. 2018; Evans and Mizrahi 2015; Forstner et al. 2007; Gruber et al. 2015; Parachin et al. 2012; Schnappinger and Ehrt 2014; Terpe 2006; Valero 2012). Prominent classical examples are the well-known LacI-, TetR-, and AraC-dependent systems (as well as optimized or modified versions thereof) for inducible expression by addition of the respective inducer. For acetic acid bacteria (AAB), including the most frequently used and studied genera *Acetobacter*, *Gluconobacter*, *Gluconacetobacter*, *Komagataeibacter*, and *Acidiphilium*, a low-cost, tight, and strongly inducible expression system has not been reported yet in the literature to the best of our knowledge. Apparently, for the heterologous systems, leakiness is a major issue responsible for the relatively low induction ratios (induced/non-induced) in AAB. In *Komagataeibacter rhaeticus* iGEM, the TetR-dependent system from transposon Tn*10* with the inducible promoter P_*tet*_ exhibited approximately only 1.5-fold induction due to high leakiness in the absence of the inducer anhydrotetracycline (Florea et al. 2016). The native l-arabinose-inducible AraC-dependent P_*BAD*_ system from *E. coli* exhibited approximately only 5- to 12-fold induction in *Gluconacetobacter xylinus* ATCC 700178, *Gluconacetobacter hansenii* ATCC 53582, and *Komagataeibacter rhaeticus* iGEM due to high basal expression and required a high concentration (4%, *w*/*v*) of the inducer l-arabinose (Teh et al. 2019). The performance of IPTG-inducible *Lac*I-dependent expression has not been reported yet for AAB according to the literature.

The AAB *G. oxydans* is industrially used for oxidative biotransformations of carbohydrates to produce, e.g., l-sorbose, a precursor in vitamin C production, dihydroxyacetone, a substance used for tanning lotions, or 6-amino-l-sorbose, a precursor of the antidiabetic drug miglitol (Ameyama et al. 1981; Gupta et al. 2001; Hekmat et al. 2003; Saito et al. 1997; Tkac et al. 2001; Wang et al. 2016). The beneficial ability of *G. oxydans* is the regio- and stereoselective incomplete oxidation of a variety of substrates (e.g., sugars and sugar alcohols) in the periplasm by membrane-bound dehydrogenases (mDHs) and release of resulting products into the cultivation medium (Mamlouk and Gullo 2013; Mientus et al. 2017; Pappenberger and Hohmann 2014). For the fully functional expression of mDHs in *G. oxydans*, the promoters of the alcohol dehydrogenase (P_GOX1067-68_) and the inositol dehydrogenase (P_GOX1857_) have been used in shuttle vectors (Mientus et al. 2017). While P_GOX1857_ is repressed in the presence of glucose (Hölscher et al. 2007), P_GOX1067-68_ showed constitutive activity with good expression (Mientus et al. 2017). Other *G. oxydans* promoters classified as strong, moderate, and weak are the constitutive promoters P_GOX0264_, P_GOX0452_, and P_GOX0384_ from genes encoding ribosomal proteins (Kallnik et al. 2010). Expression from moderate P_GOX0452_ has been used to successfully produce membrane-bound PQQ-dependent glucose dehydrogenase (GOX0265) for purification and characterization (Meyer et al. 2013). Expression from weak P_GOX0384_ has been used to successfully produce the succinate dehydrogenase from *Acetobacter pasteurianus* in *G. oxydans* as a first step toward a complete tricarboxylic acid cycle (Kiefler et al. 2015). In earlier work with *G. oxydans*, constitutive P_*tufB*_ from *G. oxydans* and from *E. coli* as well as P_*lac*_ from *E. coli* have been used (Merfort et al. 2006a, b; Schleyer et al. 2008; Tonouchi et al. 2003; Zhang et al. 2010). Since there is no Lac repressor homolog in *G. oxydans*, also P_*lac*_ is constitutive in *G. oxydans*. Together, the resulting expression plasmids with all these promoters do not allow for gradually induced expression of target genes at a desired time.

Therefore, to provide for *G. oxydans* a tight and strongly inducible plasmid, we tested AraC-P_*BAD*_ from the *E. coli* K12 derivative MC4100 in a pBBR1-based vector. The plasmids constructed in this study exhibited very low basal reporter gene expression and l-arabinose-dependent induction ratios up to 480-fold. GC-TOF-MS analysis confirmed oxidation of the inducer l-arabinose to l-arabinonic acid contributing to the acidification of the growth medium in shake flasks. This additional acidification turned out to be critical for the activity of intracellular reporter proteins in the stationary phase and could be eliminated by mDH deletion or pH-controlled conditions.

## Materials and methods

### Bacterial strains, plasmids, and culture conditions

Strains and plasmids used or created in this study are listed in Table 1. *G. oxydans* strains were routinely cultivated at 30 °C and 180 rpm in d-mannitol medium containing 4% (*w*/*v*) d-mannitol, 5 g L^-1^ yeast extract, 1 g L^-1^ KH_2_PO_4_, 1 g L^-1^ (NH_4_)_2_SO_4_, and 2.5 g L^-1^ MgSO_4_ × 7H_2_O and supplemented with 50 μg mL^-1^ cefoxitin. Unless stated otherwise, for shake flask cultivations, 50 mL of d-mannitol medium in 500 mL shaking flasks with three baffles was inoculated from overnight starter cultures to an initial optical density at 600 nm (OD_600_) of 0.3 (UV-1800, Shimadzu). If required for induction tests, l-arabinonic acid was supplemented as Li^+^ salt directly to the medium. The pH of the supplemented medium was adjusted to pH 6, cold sterile filtered and directly used for cultivation. Cultivations of *G. oxydans* harboring pBBR1MCS-2- or pBBR1MCS-5-based plasmids were supplemented with 50 μg mL^-1^ kanamycin or 10 μg mL^-1^ gentamicin, respectively (Kovach et al. 1995). *Escherichia coli* strains were routinely cultivated in lysogeny broth (LB) medium at 37 °C and 160 rpm. If appropriate, 50 μg mL^-1^ kanamycin or 10 μg mL^-1^ gentamicin was added to the medium. *G. oxydans* was transformed by conjugation using *E. coli* S17-1 as a donor (Kiefler et al. 2017). All *E. coli* strains were made competent and transformed by CaCl_2_ procedure (Hanahan 1983).

**Table 1:** Strains and plasmids used or constructed in this study

	Relevant characteristics	Reference/source
Strain		
*E. coli* DH5α	*supE*44, Δ*lacU*169 (Φ80*lacZ*ΔM15), *hsdR*17 (r_K_^−^ m_K_^+^), *recA1*, *endA1*, *gyrA96*, *thi-1*, *relA1*	Hanahan (1983)
*E. coli* S17-1	Δ*recA*, *endA1*, *hsdR17*, *supE44*, *thi*-1, *tra*^+^	Simon et al. (1983)
*G. oxydans* 621H	DSM 2343	DSMZ
*G. oxydans* BP.6	621H derivative carrying Δ*upp* and gene deletions for six mDHs	Peters et al. (2013)
Plasmid		
pBAD/Myc-His A	Vector for dose-dependent expression of recombinant proteins containing a 6×His tag in *E. coli*	Invitrogen
pBBR1MCS-2	Derivative of pBBR1MCS; Km^R^	Kovach et al. (1995)
pBBR1MCS-2-*araC*-P_*BAD*_*-uidA*	Derivative of pBBR1MCS-2 carrying reporter gene *uidA* controlled by l-arabinose-induced promoter P_*BAD*_ and *araC* encoding P_*BAD*_ regulator AraC	This work
pBBR1MCS-2-*araC*-P_*BAD*_*-mNG*	Derivative of pBBR1MCS-2-*araC*-P_*BAD*_*-uidA* using reporter gene *mNG* instead of *uidA*	This work
pBBR1MCS-2-P_GOX0264_*-mNG*	Derivative of pBBR1MCS-2 carrying reporter gene *mNG* controlled by the strong constitutive promoter of GOX0264	This work
pBBR1MCS-5	Derivative of pBBR1MCS; Gm^R^	Kovach et al. (1995)
pBBR1MCS-5-*araC*-P_*BAD*_*-uidA*	Derivative of pBBR1MCS-5 carrying reporter gene *uidA* controlled by l-arabinose-induced promoter P_*BAD*_ and *araC* encoding P_*BAD*_ regulator AraC	This work
pBBR1MCS-5-*araC*-P_*BAD*_*-mNG*	Derivative of pBBR1MCS-5-*araC*-P_*BAD*_*-uidA* using reporter gene *mNG* instead of *uidA*	This work
pBBR1MCS-5-P_*BAD*_*-mNG*	Derivative of pBBR1MCS-5-*araC*-P_*BAD*_*-mNG*, lacking the regulator gene *araC* and carrying the terminator of *gdhM* (GOX0265) upstream of P_*BAD*_	This work
pBBR1MCS-5-*araE-araC*-P_*BAD*_*-mNG*	Derivative of pBBR1MCS-5-*araC*-P_*BAD*_*-mNG* carrying gene *araE* encoding l-arabinose transporter AraE	This work
pBBR1MCS-5-*araC*-P_*BAD*_*-*MCS	pBBR1MCS-5-based empty vector for AraC-P_*BAD*_-dependent expression of target genes cloned into the MCS provided	This work
pBBR1MCS-5-*araC*-P_*BAD*_*-*MCS-*mNG*	Derivative of pBBR1MCS-5-*araC*-P_*BAD*_*-*MCS with *mNG* as reporter gene inserted into the MCS when using the restriction enzymes *Nde*I and *Xho*I	This work

### Enzymatic determination of l-arabinose concentrations

l-Arabinose concentrations in the medium of the *G. oxydans* strains 621H and BP.6 were determined using the enzymatic l-arabinose and d-galactose rapid assay kit (Megazyme). All samples were measured in microplates according to the manufacturer’s instructions by monitoring NADH formation as increase in absorption at 340 nm in a multi-well reader (infinite M1000 PRO, Tecan).

### DNA microarray analysis

To analyze short-term gene expression changes in response to a pulse of 1% (*w*/*v*) l-arabinose, the transcriptomes of *G. oxydans* 621H cultivated in complex medium with 4% (*w*/*v*) d-mannitol were compared with the control. In the mid-exponential growth phase, 1% (*w*/*v*) l-arabinose was supplemented and the same volume of water in another 621H culture as a control. After 30 min of cultivation, each cell suspension was harvested by centrifugation (4500×*g*, 5 min, 4 °C). The resulting cell pellets were directly frozen in liquid nitrogen and stored at − 80 °C until RNA preparation. The preparation of RNA, cDNA synthesis, hybridization using Agilent’s 4-plex DNA microarray platform, and data analysis were carried out as described (Kranz et al. 2018).

### Recombinant DNA work

All DNA oligonucleotides used in this study were synthesized by Eurofins MWG and are listed in Table S1. All enzymes for recombinant DNA work were purchased from Thermo Scientific. DNA manipulations by polymerase chain reaction (PCR), restriction, and ligation reactions followed standard protocols (Sambrook et al. 1989). Reporter plasmids were constructed from amplified DNA fragments and the restricted broad-host vectors pBBR1MCS-2 or pBBR1MCS-5 in a one-step isothermal Gibson assembly (Gibson et al. 2009). The terminator sequence BBa_B1002 from the iGEM parts library was placed downstream of the reporter genes. For DNA amplification by PCR, Q5 polymerase was used according to the conditions recommended by the manufacturer (New England Biolabs). All DNA modifications used to obtain designed plasmids were performed with *E. coli* DH5α. Plasmids were isolated from *E. coli* using a QIAprep spin miniprep kit (Qiagen). The plasmid inserts constructed in this work were checked by DNA sequencing (Eurofins MWG).

### Construction of plasmids

Plasmid pBBR1MCS-2-*araC*-P_*BAD*_*-uidA* was constructed using the primer pairs PF1/PF2 and PF3/PF4 to generate a 1253-bp DNA fragment with *araC-*P_*BAD*_ from the commercially available plasmid pBAD/*Myc*-His A (Invitrogen/Thermo Fischer) and a 1886-bp DNA fragment with *uidA* from *E. coli* K12 genomic DNA and the terminator BBa_B1002 inserted by elongating the 5′-end of PF4. Plasmid pBBR1MCS-2-*araC*-P_*BAD*_*-mNG* was constructed using the primer pairs PF1/PF5 and PF6/PF7 to generate a 1,238 bp DNA fragment with *araC*-P_*BAD*_ and a 790 bp DNA fragment with *mNG* and terminator BBa_B1002, respectively. For insertion of the two overlapping DNA fragments into pBBR1MCS-2 by Gibson assembly (50 °C; 1 h), pBBR1MCS-2 was restricted by the endonucleases *Sac*I and *Kpn*I. Elongated 5′-ends in primers PF2/PF3 and PF5/PF6 were used to introduce downstream of P_*BAD*_ and 6 bp upstream of the start codon of *mNG* or *uidA*, the artificial Shine-Dalgarno sequence AGGAGA (Hentschel et al. 2013). To change the plasmid backbone from pBBR1MCS-2 to pBBR1MCS-5, *araC*-P_*BAD*_*-uidA* and *araC*-P_*BAD*_*-mNG* were excised and ligated into pBBR1MCS-5 using *Sac*I and *Eco*81I.

The plasmid pBBR1MCS-5-P_*BAD*_*-mNG* was constructed using the primer pair PF8/PF9 to obtain a 1164-bp DNA fragment P_*BAD*_*-mNG* from pBBR1MCS-5-*araC*-P_*BAD*_*-mNG*. The resulting fragment was cloned into pBBR1MCS-5 using *Bam*HI and *Xho*I. To prevent undesired transcripts initiating from P_*BAD*_, the *G. oxydans* terminator of *gdhM* (GOX0265) was inserted upstream of P_*BAD*_.

Plasmid pBBR1MCS-5-*araE-araC*-P_*BAD*_*-mNG* was constructed by insertion of *araE* into pBBR1MCS-5*-araC*-P_*BAD*_*-mNG.* For that, pBBR1MCS-5*-araC*-P_*BAD*_*-mNG* was restricted by the enzymes *Asc*I and *Eco*81I and a 1576-bp DNA fragment comprising *araE* without own promoter, amplified with the primer pair PF10/PF11 from *E. coli* K12 genomic DNA, was integrated downstream of *araC* by Gibson assembly. PF10 and PF11 contained elongated 5′-ends to insert the T7 terminator sequence downstream of *araE* and a Shine-Dalgarno sequence upstream of *araE* between *araE* and *araC*. Thus, *araE* and *araC* are expected to be expressed as a polycistronic transcript from the *araC* promoter.

Plasmid pBBR1MCS-5-*araC*-P_*BAD*_-MCS as an empty vector with a ribosome-binding site, a new *Nde*I site upstream of the multiple cloning site (MCS) and the iGEM terminator sequence BBa_B1002 was constructed in two steps. In the first step, the DNA fragment *araC*-P_*BAD*_ generated with the primer pair PF12/PF13 was inserted into *Bsh*TI/*Sac*I-restricted pBBR1MCS-5 by Gibson assembly keeping the original MCS from pBBR1MCS-5. In the second step, the terminator sequence BBa_B1002 was integrated downstream of the MCS by integration of a DNA fragment amplified with the primer pair PF14/PF15. For the integration of the terminator sequence, the plasmid obtained in step one was restricted with *Xho*I and *Sph*I.

Plasmid pBBR1MCS-5-*araC*-P_*BAD*_-MCS-*mNG* was constructed from pBBR1MCS-5 by integration of the DNA fragments *araC*-P_*BAD*_ and *mNG*-BBa_B1002 in a Gibson assembly. In the amplification of the DNA fragments, the restriction sites *Nde*I and *Xho*I were integrated resulting in the same sequence as it would have been obtained *via* classical restriction cloning of *mNG* into pBBR1MCS-5-*araC*-P_*BAD*_-MCS using the restriction enzymes *Nde*I and *Xho*I. The DNA fragments *araC*-P_*BAD*_ and *mNG*-BBa_B1002 were amplified with the primer pairs PF1/PF16 and PF17/PF18, respectively. For integration, pBBR1MCS-5 was restricted using the restriction enzymes *Bsh*TI and *Kpn*I.

### Measurements of fluorescence protein and enzyme activity

For online monitoring of expression induction and relative promoter strengths of promoter–reporter constructs in *G. oxydans*, the fluorescence protein mNeonGreen (mNG) was used (Shaner et al. 2013). In shake flask experiments, *mNG* expression in *G. oxydans* was induced by addition of 1% (*w*/*v*) l-arabinose from a 50% (*w*/*v*) stock solution. Reference cultures were supplemented with an equal volume of water. In intervals, growth (OD_600_) and fluorescence emission was monitored by a spectrophotometer (UV-1800, Shimadzu) and a Tecan Reader (*λ*_ex_ 504 nm/*λ*_em_ 517 nm; gain 60; ex/em bandwidth 5 nm; infinite M1000 PRO, Tecan), respectively. In BioLector cultivations using 48-well Flowerplates® (m2p-labs), batches of 800 μL of d-mannitol medium were inoculated from overnight precultures and incubated (1200 rpm; 85% humidity, 30 °C). Over a period of up to 30 h, cell growth and fluorescence was monitored in each well simultaneously by measuring the backscatter (A_620 nm_; gain 20) and fluorescence emission (*λ*_ex_ 510 nm/*λ*_em_ 532 nm; gain 60). Typically, mean values and standard deviations of all experiments were derived from at least three biological replicates.

For enzymatic reporter assays, β-d-glucuronidase (UidA) was used (Kallnik et al. 2010). UidA activity was determined in a Miller assay essentially as described (Miller 1992). Immediately after inoculation of *G. oxydans* shake flask cultures, *uidA* expression was induced by the addition of 1% (*w*/*v*) l-arabinose. Non-induced cultures supplemented with an equal volume of water were used as controls. In intervals, 500 μL samples were taken to determine OD_600_ and UidA activity. For permeabilization of the cells, 30 μL of the culture broth in appropriate dilutions in assay buffer was incubated in a 96-well plate (20 min; 30 °C) with 100 μL of prewarmed Z-mix (54.6 mM Na_2_HPO_4_ × 7H_2_O, 36.4 mM NaH_2_PO_4_ × H_2_O, 9.1 mM KCl, 0.9 mM MgSO_4_, 45.5 mM DTT, 4.5% (*v*/*v*) chloroform, 0.5% (*w*/*v*) SDS, pH 7). After the addition of 100 μL of prewarmed 4-nitrophenyl-β-d-glucopyranoside (4 g L^-1^) using a multichannel pipette, β-d-glucuronidase activity was monitored measuring the absorption of *p*-nitrophenol in intervals of 1 min at 420 nm (infinite M1000 PRO, Tecan). From all constructs, at least three biological replicates were measured in triplicates to determine mean values and standard deviations.

### Cell flow cytometer analysis

For single cell analysis of reporter gene expression, the *G. oxydans* strains 621H and BP.6 carrying the plasmid pBBR1MCS-5-*araC-*P_*BAD*_*-mNG* were analyzed using a BD FACSAria II cell sorter (BD Biosciences) equipped with a 70-μm nozzle run with a sheath pressure of 70 psi. Using the 488 nm laser beam, the front scatter (FSC) and side scatter (SSC) were recorded as small-angle (axial) and perpendicular scatter, respectively. Combining a 502-nm-long-pass and 530/30 nm band-pass filter, the emitted mNG fluorescence from the SSC signal was detected. Fluorescence data were acquired using a two-step gating strategy: at first, signals from cell debris and electronic noise were excluded by gating a population in a FSC-H vs. an SSC-H plot. Secondly, to perform singlet discrimination, from the resulting population the FSC-H signal was plotted against FSC-W. The gated singlet population was used for fluorescence acquisition in all experiments. While the total event rate during measurements never exceeded 15,000 events/s, for each sample, the signals of 100,000 events were recorded. For FACS device control and data analysis, FACSDiva 7.0.1 software (BD Biosciences) was used. Gated events (*n* = 100,000) were used for data analysis in FlowJo for Windows 10.4.2 (FlowJo, LLC) and Prism 7.04 (GraphPad Software) to visualize FACS data.

### Bioreactor cultivation of *G. oxydans*

Bioreactor cultivations were conducted in DASbox® mini-bioreactors controlled by DASware software (Eppendorf). In 385-mL glass vessels equipped with two 6-bladed Rushton-type impellers, O_2_ (InPro® 6800 series, Mettler-Toledo), pH (EasyFerm Plus K8 120, Hamilton), and temperature sensors, 150-mL 4% (*w*/*v*) d-mannitol medium was inoculated to an initial OD_600_ of 0.3 (30 °C). The initial gas flow rate was set to 6 sL h^−1^. Starting agitation frequency was 500 rpm. The pH was maintained at 6 by automatic titration using H_2_SO_4_ or KOH stocks (1.5 M). Dissolved oxygen tension in the medium was maintained ≥ 30% by a cascade of first adjusting the agitation speed to a maximum of 1200 rpm, then by rising the O_2_ concentration in the supplied gas up to 80% (*v*/*v*) and eventually by increasing the gas flow rate. In intervals, samples were taken from the culture broth and OD_600_ as well as fluorescence were determined as described above.

### l-Arabinose biotransformation for the analysis of oxidation products

To identify the reaction products of l-arabinose oxidation by *G. oxydans* strains, cells were grown in 25 mL of d-mannitol medium in 500 mL shake flasks to an OD_600_ of 1.6, centrifuged (4000×*g*, 5 min) and washed twice with 1 mM HEPES (pH 7). The pellet was resuspended in 25 mL biotransformation buffer (33.9 g L^-1^ Na_2_HPO_4_, 15 g L^-1^ KH_2_PO_4_, 5 g L^-1^ NH_4_Cl, 2.5 g L^-1^ NaCl, 0.49 g L^-1^ MgSO_4_, 0.02 g L^-1^ CaCl_2_) supplemented with 1% (*w*/*v*) l-arabinose. The cell suspensions were incubated for 24 h at 30 °C on a shaker (180 rpm). Afterwards, cells were centrifuged (4000×*g*, 5 min) and the cell-free supernatant was analyzed using a gas chromatograph (Agilent 6890N, Agilent Technologies) coupled to a Waters Micromass GCT Premier high-resolution time-of-flight mass spectrometer (Waters). Sample derivatization, GC-TOF-MS operation, and peak identification were essentially conducted as described (Paczia et al. 2012). Biotransformation medium without cells was used as reference.

## Results

### Growth of *G. oxydans* 621H in the presence of l-arabinose

**Fig. 1:**
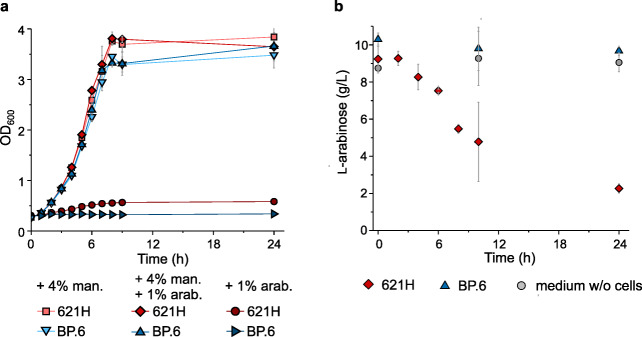
Comparisons of the *G. oxydans* strains 621H and BP.6. **a** Growth (OD_600_) in shake flasks in complex medium with 4% (*w*/*v*) d-mannitol or 1% (*w*/*v*) l-arabinose as well as both 4% (*w*/*v*) d-mannitol plus 1% (*w*/*v*) l-arabinose. **b** Arabinose concentrations in complex medium with 4% (*w*/*v*) d-mannitol plus 1% (*w*/*v*) l-arabinose in shake flasks. Data represent mean ± SD from three biological replicates

In the present study, we wanted to test l-arabinose-dependent gene expression in *G. oxydans* 621H to provide a regulatable expression plasmid for this AAB, similar to AraC-dependent vectors constructed for *E. coli* (Guzman et al. 1995). In such a system, the inducer l-arabinose needs to enter the cell where it binds to the transcriptional regulator AraC (Schleif 2010). In the case of *G. oxydans* 621H, l-arabinose was reported to be oxidized already in the periplasm by the membrane-bound glucose DH GdhM (GOX0265) (Mientus et al. 2017; Peters et al. 2013). Thus, the presence of l-arabinose could affect the growth of *G. oxydans* 621H by using it as an energy source through oxidation, thereby inactivating the inducer. The *G. oxydans* multi-deletion strain BP.6 lacks GdhM and exhibited no l-arabinose-oxidizing activity anymore (Peters et al. 2013). Therefore, we first tested the impact of l-arabinose on growth of the *G. oxydans* strains 621H and BP.6 in shake flasks. In complex medium supplemented with 1% (*w*/*v*) l-arabinose instead of d-mannitol, growth of *G. oxydans* 621H was very poor and OD_600_ merely doubled within 8 h, while strain BP.6 did not grow at all (Fig. 1a). In medium supplemented with 4% (*w*/*v*) d-mannitol plus 1% (*w*/*v*) l-arabinose, both strains grew very similar (*μ* = 0.18 ± 0.01 h^−1^ for 621H and 0.17 ± 0.01 h^−1^ for BP.6) compared with the control condition with 4% (*w*/*v*) d-mannitol, yet without arabinose (*μ* = 0.18 ± 0.01 h^-1^ for 621H and 0.17 ± 0.01 h^−1^ for BP.6). In the stationary phase, both strains reached similar final OD_600_ values independent of the l-arabinose supplementation (621H OD_600_ with d-mannitol 3.84 ± 0.18 and with d-mannitol + l-arabinose 3.65 ± 0.28; BP.6 OD_600_ with d-mannitol 3.48 ± 0.26 and with d-mannitol + l-arabinose 3.66 ± 0.08). The concentration of l-arabinose supplemented to the medium did not decrease without cells or with BP.6 cells, while in the 621H cultures l-arabinose was decreased by approximately 80% within 24 h (Fig. 1b). This suggested that l-arabinose or its oxidation product did not impair growth of *G. oxydans*.

### l-Arabinose is oxidized to l-arabinonic acid and hardly affected global gene expression

Based on the measurement of whole cell l-arabinose-oxidation activities of *G. oxydans* mDH deletion strains and of specifically complemented strains using DCPIP as electron acceptor, the membrane-bound glucose DH is expected to be responsible for l-arabinose oxidation (Mientus et al. 2017; Peters et al. 2013). Therefore, we tested the *G. oxydans* strains 621H and BP.6 for L-arabinose oxidation and analyzed the product(s) by GC-TOF-MS analysis. The multi-deletion strain BP.6 lacks six mDHs including the membrane-bound glucose DH GdhM (GOX0265) and still possesses the cytosolic glucose DH (GOX2015) GdhS (Peters et al. 2013). Cell suspensions of each strain with an OD_600_ of 1.6 were incubated for 24 h at 30 °C and 180 rpm in biotransformation buffer supplemented with 1% (*w*/*v*) l-arabinose. Afterwards, cell-free supernatants were obtained for GC-TOF-MS measurements. Strain 621H clearly oxidized l-arabinose, and l-arabinonic acid was identified as the product, while strain BP.6 consumed only minor amounts of l-arabinose and formed minor amounts of l-arabinonic acid (Fig. S1). The 621H sample showed two new peaks at retention times (*R*_t_) 13.49 and 14.74 min. The peak at *R*_t_ 13.49 min had an *m*/*z* value of 364, corresponding to arabinonic acid γ-1,4-lactone with 3 TMS groups. The peak at *R*_t_ 14.74 min had an *m*/*z* value of 511, corresponding to arabinonic acid with 5 TMS groups and a methyl group split off. According to the comparable arbitrary peak areas, data indicated that GdhM did significantly contribute to l-arabinose oxidation. For strain BP.6, only a very low amount of l-arabinonic acid was found (~ 3%) compared with 621H. Together, this indicated that l-arabinose was indeed mainly oxidized by the membrane-bound glucose DH, while the contribution of the cytosolic glucose DH GdhS or another enzyme remaining in strain BP.6 was negligible.

To check whether the addition of l-arabinose as an inducer possibly affects short-term global gene expression in *G. oxydans* 621H, transcriptomes were compared using DNA microarrays. Addition of 1% (*w*/*v*) l-arabinose in the mid-exponential growth phase to 621H cells cultured in complex medium with d-mannitol had only very little effects on the relative mRNA levels within 30 min (Table S2). The highest mRNA level increase (2-fold) was found for GOX0707 encoding the DNA protection during starvation protein Dps followed by six hypothetical proteins (1.6- to 1.9-fold). The strongest mRNA level decrease (0.46-fold) was observed for GOX0536 encoding a hydroxamate-type ferrisiderophore receptor followed by its neighboring genes GOX0532 (0.57-fold) and GOX0531 (0.59-fold) encoding the ExbB and ExbD proteins of the TonB-ExbB-ExbD system as well as GOX0758 (0.56-fold) encoding a porin protein. There is no obvious functional link between these genes and l-arabinose metabolism.

### Construction of AraC-dependent l-arabinose-inducible expression plasmids

Based on the tight high-level expression vectors containing the P_*BAD*_ promoter developed for *E. coli* (Guzman et al. 1995), Invitrogen commercialized the AraC-P_*BAD*_ system for dose-dependent expression of genes with plasmid pBAD/Myc-His A useful for expression of potentially toxic or essential genes in *E. coli*. The *araC* sequence used in pBAD/Myc-His A corresponds to the *araC* sequence of the widely used MC4100 lineage of *E. coli* K12 (Casadaban 1976). It differs in nine codons from *araC* of the *E. coli* K12 reference strain MG1655, which could be of advantage for expression in *G. oxydans*. Six of the nine different codons cluster in the region of the helix-turn-helix motif responsible for DNA binding at the C terminus of AraC (Brunelle and Schleif 1989). Five of these six codons exhibit much higher usage frequency in *G. oxydans* when using *araC* from MC4100 instead of MG1655 (Table S3). Consequently, the AraC-dependent promoter P_*BAD*_ flanked by *araC* from MC4100 and the reporter gene *uidA* or *mNeonGreen* was integrated into the multiple cloning site (MCS) as described in “Materials and methods*.*” To enable efficient translation of the reporter, 16 nt containing a proven Shine-Dalgarno sequence functional in *G. oxydans* was placed between P_*BAD*_ and the reporter gene. Furthermore, as a terminator sequence, BBa_B1002 was inserted downstream of the reporter genes. We also constructed a reporter plasmid without *araC* to test the AraC dependence of P_*BAD*_ in *G. oxydans*. Another reporter plasmid additionally carried the l-arabinose transporter gene *araE* to test the sensitivity of induction towards l-arabinose (Fig. S2).

### Performance of the AraC-P_*BAD*_ system with enzyme reporter UidA in shake flasks

**Fig. 2:**
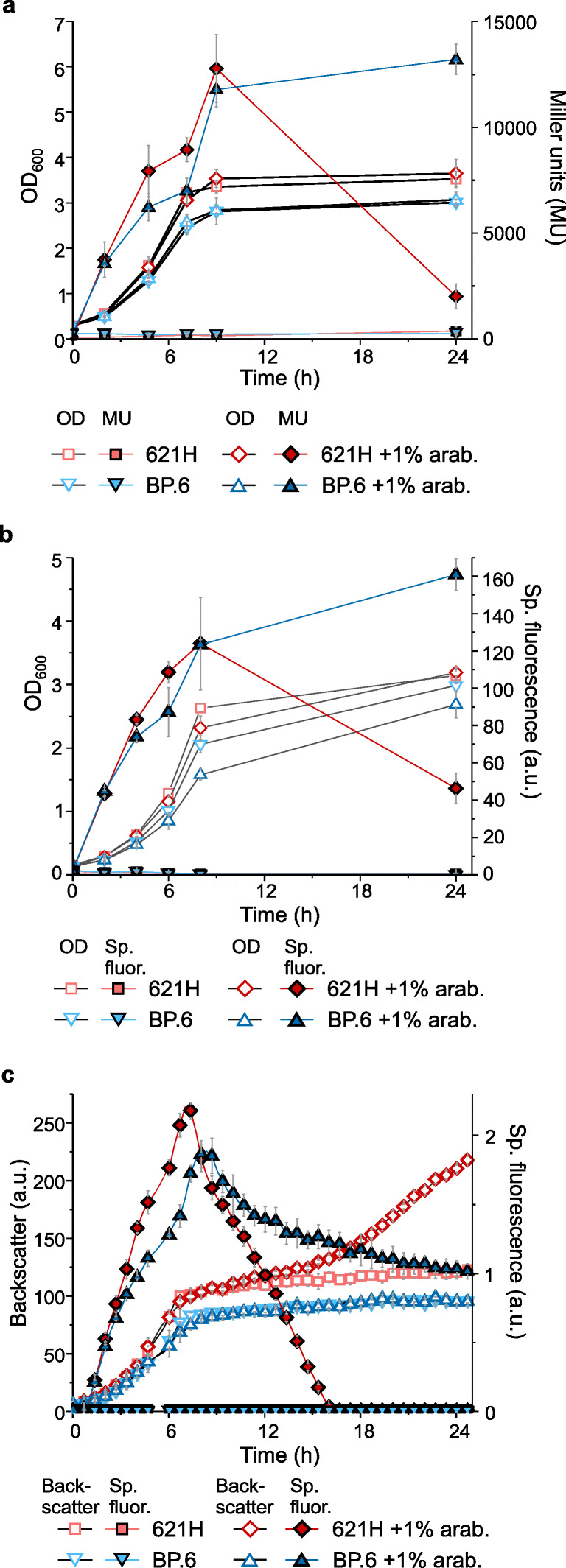
Comparisons of the *G. oxydans* strains 621H and BP.6. **a** Growth and UidA activity in Miller units (MU) in strains 621H and BP.6 carrying plasmid pBBR1MCS-5-*araC*-P_*BAD*_*-uidA* in l-arabinose-induced and non-induced condition in shake flasks. **b** Growth and specific mNeonGreen (mNG) fluorescence in strains 621H and BP.6 carrying plasmid pBBR1MCS-5-*araC*-P_*BAD*_*-mNG* in l-arabinose-induced and non-induced condition in shake flasks. The mNG fluorescence was measured in a Tecan reader. The specific fluorescence was calculated from absolute fluorescence per OD_600_. **c** Growth according to backscatter and specific mNG fluorescence in strains 621H and BP.6 carrying plasmid pBBR1MCS-5-*araC*-P_*BAD*_*-mNG* in l-arabinose-induced and non-induced condition in microscale BioLector cultivations. The specific fluorescence was calculated from absolute fluorescence per backscatter. For induction, always 1% (*w*/*v*) l-arabinose was added to the d-mannitol medium. For all experiments, data represent mean ± SD from three biological replicates

With the pBBR1MCS-5-*araC*-P_*BAD*_*-uidA* plasmid, we tested the basal expression and the induction by l-arabinose using the reporter enzyme β-d-glucuronidase. Besides *G. oxydans* 621H, which oxidizes l-arabinose by mDHs, we also used the multi-deletion strain BP.6 that is almost unable to oxidize l-arabinose to check whether AraC-P_*BAD*_ performs better in BP.6 than in 621H. Both strains were grown in shake flasks using d-mannitol medium with and without 1% (*w*/*v*) l-arabinose. For strain 621H, the highest UidA activity was observed at the end of the exponential phase after approximately 9 h with 12,772 ± 1604 MU compared with 146 ± 47 MU in the control cultures without l-arabinose, the latter indicating very low basal expression (Fig. 2a). Strain BP.6 exhibited 11,780 ± 813 MU after 9 h in induced cultures compared with 225 ± 29 MU in the non-induced controls. Surprisingly, UidA activity in l-arabinose-supplemented *G. oxydans* 621H was highly reduced after 24 h (2,007 ± 583 MU), while UidA activity in BP.6 remained high after 24 h (13,209 ± 715 MU). When compared with the non-induced cultures, maximal UidA induction ratios of 87 and 59 were calculated for 621H and BP.6, respectively (Table 2). The lower ratio calculated for BP.6 was primarily due to the somewhat higher basal expression under non-induced condition, while the absolute UidA activity in BP.6 under induced conditions was similar as that in 621H. Generally, the UidA activities in the non-induced cultures did barely surpass the background activity values determined in cell-free control samples. This indicated that the expression plasmid pBBR1MCS-5-*araC*-P_*BAD*_*-uidA* showed very low basal expression of P_*BAD*_ in the absence of l-arabinose.

**Table 2:** l-Arabinose-dependent induction fold changes calculated from UidA activities in *G oxydans* strains 621H and BP.6 carrying plasmid pBBR1MCS-5-*araC*-P_*BAD*_*-uidA*

Time (h)	621H	BP.6
2	62 ± 30	16 ± 5
4	93 ± 67	35 ± 7
7	50 ± 7	34 ± 12
9	98 ± 46	53 ± 3
24	7 ± 4	52 ± 3

Cells were cultivated in shake flasks in d-mannitol medium without and with 1% (*w*/*v*) l-arabinose for induction (Fig. 2a). Data represent mean ± SD from three biological replicates

### Performance of the AraC-P_*BAD*_ system with the fluorescence reporter protein mNeonGreen

For continuous determination of reporter gene expression and its dynamics as well as to verify the strong induction of P_*BAD*_ and AraC dependence in *G. oxydans*, we also tested the fluorescence reporter protein mNeonGreen (mNG). In all tests with plasmid pBBR1MCS-5-*araC*-P_*BAD*_*-mNG*, the mNG fluorescence signals in non-induced cultures did barely surpass the background signals of cell-free control samples, suggesting also a very low basal expression of *mNG*. In shake flask cultivations with 1% (*w*/*v*) l-arabinose, the mNG fluorescence in *G. oxydans* 621H peaked approximately after 8 h (Fig. 2b). At the end of the cultivation (24 h), the mNG fluorescence was much lower in strain 621H, while in strain BP.6, the mNG fluorescence was further increased. The maximal induction ratios based on the absolute or the biomass-specific mNG fluorescence were calculated to be 289 ± 75 or 327 ± 71 for strain 621H and 431 ± 14 or 481 ± 38 for BP.6, respectively (Table 3). In microscale BioLector cultivations, a similar induction profile was observed (Fig. 2c). The maximal induction ratios based on specific fluorescence were calculated to be 222 ± 8 for 621H and 192 ± 8 for BP.6. Again, after entering the stationary phase, the specific mNG fluorescence steadily decreased and reached zero after about 16 h in strain 621H, while for strain BP.6, the decrease was much weaker reduced over time and remained high. This was in contrast with the shake flask cultivations, where mNG fluorescence increased in the stationary phase in strain BP.6 (Fig. 2b). In the late stationary phase (16–24 h), the backscatter values of the 621H cultures supplemented with 1% (*w*/*v*) l-arabinose steadily increased further. Measurements of the OD_600_ in a photometer showed no differences between l-arabinose-supplemented strain 621H and the non-supplemented 621H cultures. Additional control measurements with cell-free complex medium adjusted to different pH values (pH 6, 4.5, 4, and 3.3) revealed differences in backscatters already in cell-free medium due to the differences in pH that could not be observed in OD_600_ measurements in a photometer. This suggested that the steady increase of backscatter values in l-arabinose-induced 621H cultures in the stationary phase resulted from a stronger acidification of the growth medium that cannot be detected by OD_600_ measurements in a photometer. Taken together, the newly constructed expression plasmids for *G. oxydans* based on the AraC-P_*BAD*_ system exhibited very low basal expression of the reporter proteins UidA and mNG and very strong induction by 1% (*w*/*v*) l-arabinose both in shake flask and in microscale BioLector cultivations (up to 480-fold).

**Table 3:** l-Arabinose-dependent induction fold changes calculated from mNG signals in *G. oxydans* strains 621H and BP.6 carrying plasmid pBBR1MCS-5-*araC*-P_*BAD*_*-mNG*

Time (h)	abs. fluorescence-fold		sp. fluorescence-fold	
	621H	BP.6	621H	BP.6
2	38 ± 4	31 ± 4	39 ± 2	32 ± 3
4	51 ± 4	35 ± 2	53 ± 3	38 ± 2
6	126 ± 12	73 ± 9	140 ± 13	88 ± 14
8	289 ± 75	194 ± 8	327 ± 71	254 ± 16
24	128 ± 61	431 ± 14	125 ± 58	481 ± 38
30	45 ± 15	327 ± 20	48 ± 16	392 ± 28

Cells were cultivated in shake flasks in d-mannitol medium with and without 1% (*w*/*v*) l-arabinose for induction (Fig. 2b). Fold changes (1% l-arabinose vs. no inducer) were calculated from absolute mNG fluorescence signals (abs.) and from backscatter-related biomass-specific mNG fluorescence signals (sp.). Data represent mean ± SD from three biological replicates

### The P_*BAD*_ promoter is AraC-dependent in *G. oxydans* and tunable by varying the l-arabinose concentrations

In *E. coli*, the regulator AraC acts as a repressor of P_*BAD*_ in the absence of l-arabinose by bending the promoter DNA and stimulates transcription from P_*BAD*_ when l-arabinose is present (Schleif 2010; Soisson et al. 1997). To rule out the possibility that in *G. oxydans* P_*BAD*_ is either repressed or activated by a *G. oxydans* protein, the plasmid pBBR1MCS-5-P_*BAD*_*-mNG* missing the *araC* gene was constructed and tested in *G. oxydans* 621H. In this experiment, no differences in fluorescence were observed between induced and non-induced cells, thus no induction was observed with 1% (*w*/*v*) l-arabinose when AraC was absent (Fig. S3). This result showed that the inducibility of P_*BAD*_ in *G. oxydans* is independent of endogenous proteins and indeed specifically dependent on heterologous AraC. Furthermore, in the absence of AraC the reporter is almost not expressed from P_*BAD*_, thus repression of P_*BAD*_ by AraC seemed not to be required in *G. oxydans*.

**Fig. 3:**
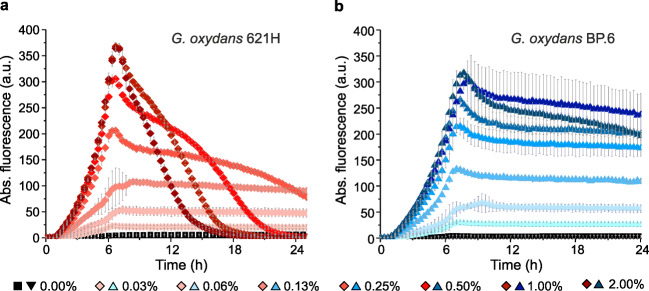
l-Arabinose-dependent modulation of expression in the *G. oxydans* strains 621H (**a**) and BP.6 (**b**) carrying plasmid pBBR1MCS-5-*araC-*P_*BAD*_*-mNG* in microscale BioLector cultivations. Reporter gene mNG expression was induced with increasing concentrations of l-arabinose from 0.03 to 2% (*w*/*v*) as indicated. Data represent mean ± SD from three biological replicates

The tunability of the AraC-P_*BAD*_ system in *G. oxydans* was tested with a range of l-arabinose concentrations. The mNG fluorescence gradually increased similarly in strains 621H and BP.6 with increasing concentrations of l-arabinose (Fig. 3). Compared with 1% (*w*/*v*) l-arabinose, supplementation with 2% (*w*/*v*) l-arabinose did not lead to a significant increase in mNG fluorescence, indicating saturation of induction at close to 1% (*w*/*v*) inducer. Whereas only a small decrease of the mNG fluorescence was observed in the stationary phase for the BP.6 cultures induced with the highest l-arabinose concentrations, mNG fluorescence for strain 621H decreased to zero in the stationary phase for the cultures induced with more than 0.5% (*w*/*v*) l-arabinose. In contrast, no decrease was observed for the 621H cultures with up to 0.25% l-arabinose, clearly suggesting a correlation between l-arabinonic acid formation and loss in mNG fluorescence signals.

Overall, in the exponential growth phase, the performance of the AraC-P_*BAD*_ system was very similar in the l-arabinose-oxidizing strain 621H compared with the multi-deletion strain BP.6. Therefore, we asked whether the oxidation product l-arabinonic acid could possibly also act as an inducer on AraC, thereby compensating for the decrease in l-arabinose by its oxidation in 621H cultures. To test this, we added 1% (*w*/*v*) of l-arabinonic acid to mannitol medium and monitored the mNG fluorescence of strain 621H with pBBR1MCS-5-*araC*-P_*BAD*_*-mNG* in BioLector cultivations. l-Arabinonic acid did not induce the AraC-P_*BAD*_ system, since the cultures exhibited a similar background fluorescence as non-induced 621H cultures without l-arabinose or l-arabinonic acid (Fig. S4).

The experiments described above showed that l-arabinose is able to activate AraC in *G. oxydans*, yet it is unknown how l-arabinose enters the *G. oxydans* cytoplasm. Knowledge on sugar transport systems in *G. oxydans* is scarce in general and no data are available for l-arabinose to our knowledge. The *E. coli* protein AraE is a low-affinity high-capacity l-arabinose transporter (Khlebnikov et al. 2001). To facilitate l-arabinose uptake by *G. oxydans* and thereby possibly improving the sensitivity of the l-arabinose-inducible system, plasmid pBBR1MCS-5-*araE-araC*-P_*BAD*_*-mNG* was constructed containing *araE* including a Shine-Dalgarno sequence directly downstream of *araC*. This should enable co-transcription of *araC* and *araE* from P_*araC*_. When analyzing strain 621H carrying plasmid pBBR1MCS-5-*araE*-*araC*-P_*BAD*_-*mNG* with 0.1 and 0.5% (*w*/*v*) l-arabinose, however, the specific fluorescence was 40 to 60% lower compared with strain 621H carrying the plasmid without *araE* (Fig. S5). Thus, no increase in sensitivity towards the inducer was observed with pBBR1MCS-5-*araE*-*araC*-P_*BAD*_-*mNG* under the conditions tested.

### Acidification of the growth medium is responsible for the decrease in reporter activities

In the experiments described above, both UidA activity and mNG fluorescence strongly decreased in the stationary phase of the 621H cultures induced with 1% (*w*/*v*) l-arabinose but not in the BP.6 cultures. The decrease correlated with the l-arabinose concentration and was absent at concentrations up to 0.25% (*w*/*v*). This clearly indicated that oxidation of l-arabinose affected the dynamics of the reporter activities. According to previous data and our GC-TOF-MS results, strain 621H oxidizes l-arabinose to l-arabinonic acid by the membrane-bound glucose DH, which is absent in strain BP.6 (Mientus et al. 2017). With a p*K*_a_ of 3.39 (https://hmdb.ca/metabolites/HMDB0000539), formation of l-arabinonic acid caused an additional acidification of the growth medium. The pH values of the d-mannitol media of strains 621H and BP.6 supplemented with 1% (*w*/*v*) l-arabinose decreased from initially pH 6.0 to 3.3 ± 0.1 and 4.4 ± 0.1, respectively, after 24 h. In contrast, the pH values of the non-induced cultures were 4.7 ± 0.1 and 4.6 ± 0.1 for 621H and BP.6, respectively, after 24 h. These data confirm that l-arabinose oxidation to l-arabinonic acid by strain 621H leads to a stronger acidification of the medium.

Bacteria typically exhibit energy-dependent mechanisms for cytoplasmic pH homeostasis in order to survive during exposure to acidic or alkaline conditions. The observed decreases in mNG fluorescence suggested that due to the formation of l-arabinonic acid, the cytoplasmic pH in strain 621H increasingly acidified in the stationary phase when cells starved for energy. Recently, it was shown for the mNG protein that a shift from pH 6 to 4 is sufficient to reduce the mNG fluorescence by approximately 75% (Steiert et al. 2018). As described above, the pH values of induced 621H cultures after 24 h were even lower than pH 4. Consequently, we tested whether fresh d-mannitol-free and l-arabinose-free medium adjusted to different pH values could restore the mNG fluorescence of stationary phase 621H cells cultivated with 1% (*w*/*v*) l-arabinose. For this test, we used plasmid pBBR1MCS-2-P_GOX0264_-*mNG* in order to constitutively express *mNG* from the strong promoter of GOX0264. As expected, when cultivated with l-arabinose the mNG fluorescence was clearly decreased in the stationary phase, while without l-arabinose the mNG fluorescence remained high (Fig. S6a). Incubation of the cells grown with l-arabinose for 1 h in fresh medium adjusted to pH 3.5, 4.9, and 6.2 gradually recovered the mNG fluorescence (Fig. S6b). This result indicated that the decrease in mNG fluorescence in 621H cells was hardly due to degradation of the mNG protein in the stationary phase. Rather, the strong acidification of the growth medium by l-arabinonic acid formation in 621H cultures affected the cytoplasmic pH during the stationary phase which in turn was responsible for the decrease in mNG fluorescence. Furthermore, for *G. oxydans* 621H carrying pBBR1MCS-5-*araC-*P_*BAD*_*-uidA* grown with 1% (*w*/*v*) l-arabinose, the loss of UidA activity in the stationary phase (24 h) was also restored partly when cells were transferred for 1 h into fresh medium adjusted to pH 6 (Fig. S6c, d).

**Fig. 4:**
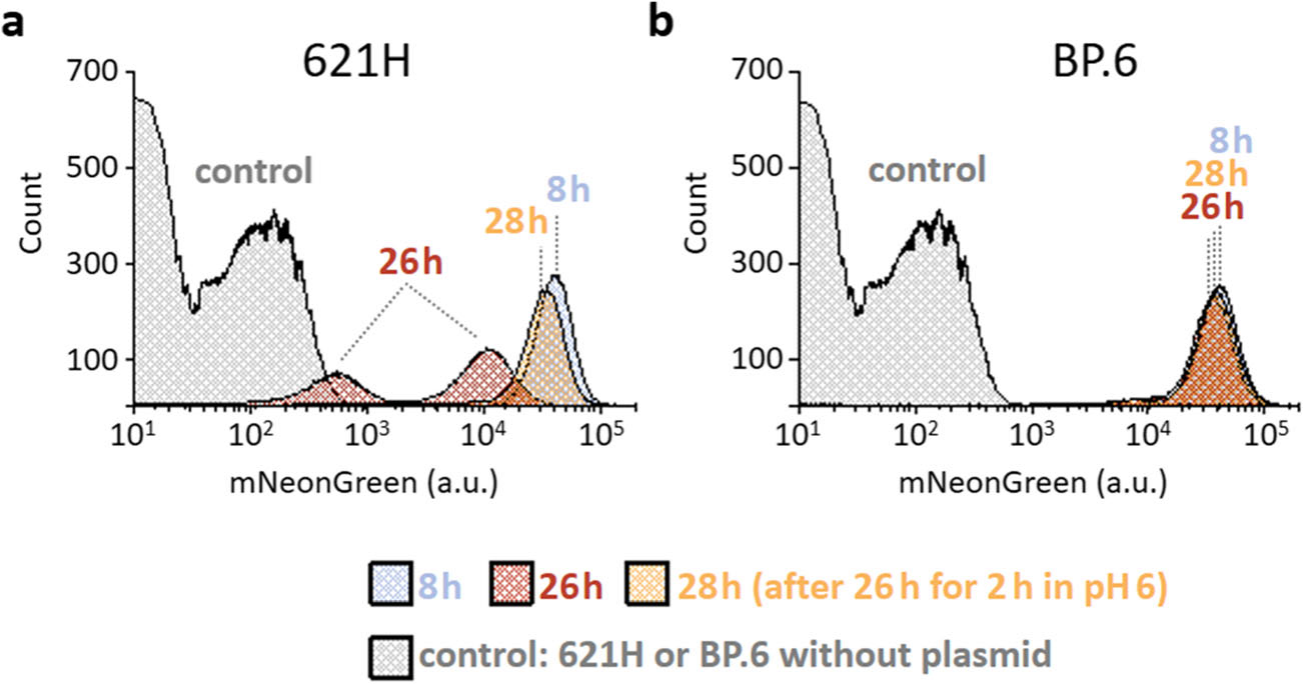
FACS analysis of the *G. oxydans* strains 621H (**a**) and BP.6 (**b**) carrying plasmid pBBR1MCS-5-*araC*-P_*BAD*_*-mNG*. Cells were grown in shake flasks with d-mannitol medium and induced with 1% (*w*/*v*) l-arabinose. FACS analysis was performed 8 and 26 h after induction. After 26 h, cells were transferred into fresh l-arabinose- and d-mannitol-free medium adjusted to pH 6 followed by incubation on a rotary shaker for 2 h. As a control, 621H or BP.6 cells without plasmids also grown in d-mannitol medium with 1% (*w*/*v*) l-arabinose were used. Total counts per sample represent 100,000 events and only appears to be different due to the logarithmic scale on the *x*-axis

To analyze the induction of the Ara*C-*P_*BAD*_ system in *G. oxydans* and the pH-dependent decrease and recovery in mNG fluorescence on the single cell level, flow cytometer analysis was applied. For strain 621H, 8 h after induction, 91% of all cells exhibited high fluorescence signals (approximately 40,000 a.u.) within the chosen gate, indicating a high population homogeneity close to the end of the exponential growth phase (Fig. 4a). 26 h after induction, when the pH of the medium was 3.3 ± 0.1, the overall fluorescence was diminished and two distinct populations at approximately 10,000 a.u. and approximately 600 a.u. emerged. Transferring the 621H cells into d-mannitol-free and l-arabinose-free medium adjusted to pH 6 resulted in a recovery of the mNG fluorescence in a single population with 83% exhibiting a fluorescence signal of approximately 30,000 a.u.. For *G. oxydans* BP.6, also a strong induction with a high homogeneity and a fluorescence of about 40,000 a.u. was observed after 8 h. These signals were hardly decreased in the stationary phase after 26 h (pH 4.4 ± 0.1) and were not affected by the transfer into fresh medium adjusted to pH 6 (Fig. 4b). These single cell results are in line with the assumption that additional acidification of the growth medium causes a stronger intracellular acidification in the stationary phase. Furthermore, the single cell data showed that the AraC-P_*BAD*_ system enabled a highly uniform induction response in the cells.

### UidA activity and mNG fluorescence are stable in pH-controlled bioreactor cultivations

**Fig. 5:**
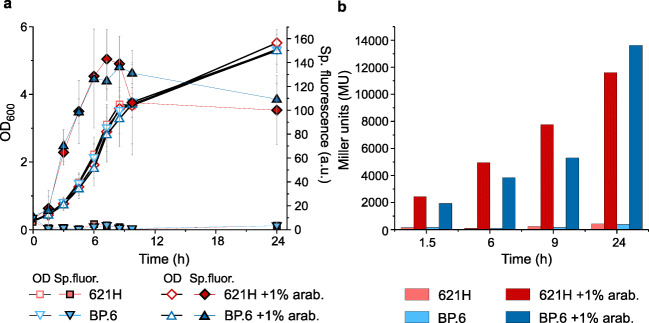
l-Arabinose-inducible reporter gene expression in DASbox fermentations in pH-controlled conditions (pH 6). Both, mNG and UidA activity remained high in *G. oxydans* 621H carrying plasmid pBBR1MCS-5-*araC*-P_*BAD*_*-mNG* (**a**) or pBBR1MCS-5-*araC*-P_*BAD*_*-uidA* (**b**) 24 h after induction with 1% (*w*/*v*) l-arabinose when pH 6 was maintained

In the following, pH-controlled bioreactor cultivations were carried out to circumvent effects due to acidification of the medium when assaying the performance of the l-arabinose-inducible AraC-P_*BAD*_-system in *G. oxydans*. Therefore, *G. oxydans* strains 621H and BP.6 carrying pBBR1MCS-5-*araC*-P_*BAD*_*-uidA* and pBBR1MCS-5-*araC*-P_*BAD*_*-mNG* were grown in DASbox® mini bioreactors under controlled conditions at pH 6 and dissolved oxygen concentrations above 30%. Sufficient oxygen levels are crucial for maturation of the chromophore (Shaner et al. 2013). After the 24-h fermentation in d-mannitol medium with 1% (*w*/*v*) l-arabinose, the total volume of potassium hydroxide solution consumed to maintain pH 6 in the 621H cultures (11.2 ± 0.7 mL) was much higher compared with the BP.6 cultures (6.0 ± 1.0 mL), which is in line with the l-arabinose oxidation by strain 621H yielding l-arabinonic acid. As expected, when maintaining a constant pH of 6 throughout the cultivation, neither the mNG fluorescence nor the UidA activity decreased in the stationary phase in 621H cells oxidizing l-arabinose (Fig. 5).

### Providing an AraC-P_*BAD*_-dependent plasmid with a multiple cloning site as empty vector

**Fig. 6:**
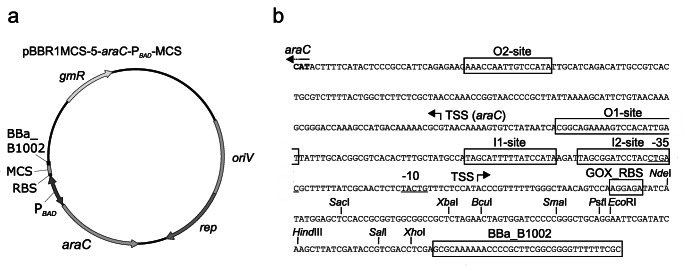
Scheme of the pBBR1MCS-5-based *araC*-P_*BAD*_ plasmid with a multiple cloning site (**a**) and sequence information details (**b**). The ribosome-binding site AGGAGA (GOX_RBS) is included and usable when the insert cloning is carried out on the 5′-end by *Nde*I; otherwise, another RBS needs to be included in the insert upstream of the gene of interest. The iGEM terminator sequence of BBa_B1002 is located downstream of the multiple cloning site (MCS)

Albeit an AraC-P_*BAD*_-dependent expression plasmid with genes of interest could be easily obtained by Gibson assembly in an all-in-one fragment mixture cloning, we wanted to provide an AraC-P_*BAD*_-dependent plasmid for *G. oxydans* with a multiple cloning site as an empty vector for classical restriction enzyme-based cloning attempts to insert genes of interest. Therefore, we assembled *araC*-P_*BAD*_ with the multiple cloning site from pBBR1MCS-5 and the terminator sequence BBa_B1002 from the iGEM parts library in plasmid pBBR1MCS-5 to obtain plasmid pBBR1MCS-5-*araC*-P_*BAD*_-MCS as an empty vector (Fig. 6). In this plasmid, the ribosome binding site AGGAGA was included upstream of the *Nde*I site newly created upstream of the original MCS by exchanging a single G to a C. To check the functionality and inducibility of AraC-P_*BAD*_ in this empty vector as a multiple cloning reference plasmid, we created the reporter plasmid pBBR1MCS-5-*araC*-P_*BAD*_-MCS-*mNG* by Gibson assembly resulting in the same sequence as it would have been obtained *via* classical restriction cloning of *mNG* using the restriction enzymes *Nde*I and *Xho*I. When the resulting plasmid pBBR1MCS-5-*araC*-P_*BAD*_-MCS-*mNG* was tested in strain 621H, again strong induction in the presence of l-arabinose was observed and the basal expression in the absence of l-arabinose was again very low (Fig. S7). However, for unknown reasons the specific mNG fluorescence reached only approximately 40% of the maximum observed with the test plasmid always used before and not having the multiple cloning site or remaining parts thereof. Nevertheless, this demonstrated that plasmid pBBR1MCS-5-*araC*-P_*BAD*_-MCS was also functional and could serve as a multiple cloning vector to insert fragments containing genes of interest and if required own ribosome binding sites.

## Discussion

For the AAB *G. oxydans*, no expression plasmid was available that shows very low background expression and allows strong and tunable target gene expression. In this study, we found that the AraC-P_*BAD*_ system derived from *E. coli* K12 MC4100 performed very well in *G. oxydans* with the pBBR1 plasmid backbone. It exhibited very low basal reporter gene expression, induction ratios up to 480-fold, and gradually increased expression in an l-arabinose concentration-dependent manner. Typically, the regulatable expression plasmids tested in AAB species and reported so far suffered from very leaky expression in the absence of the respective inducer, resulting in very low induction ratios for the promoters P_*BAD*_, P_*Tet*_ or P_*Lux*_, while the overall expression was strong according to the reporter activities (Florea et al. 2016; Teh et al. 2019). Among these three promoters, the best induction ratios were only 5- to 12-fold for the l-arabinose-inducible AraC-P_*BAD*_ system from *E. coli* when used in *G. xylinus* 700178, *G. hansenii* 53582, or *K. rhaeticus* iGEM (Teh et al. 2019). The reason for the leakiness in AAB reported for the repressor-regulated promoters is not clear. It seems the binding of the repressors to their target DNA sequences is not strong enough in the cytoplasm of AAB or there is not enough repressor protein. However, in the case of AraC the mechanism of transcriptional gene regulation is different compared with those of the pure repressor proteins TetR, LuxR, and LacI, which just dissociate from their operators when their respective inducer is bound to the protein. In *E. coli*, AraC does not only repress P_*BAD*_ by looping the DNA when bound to specific target sequences in the absence of l-arabinose but also is essential for activation of P_*BAD*_ in the presence of l-arabinose *via* a modified binding to target sequences, causing unlooping of the promoter DNA and stimulation of both the binding of RNA polymerase and the transition from the closed to the open promoter complex (Schleif 2010; Soisson et al. 1997). Additionally, in *E. coli*, the cAMP receptor protein (CRP) also plays an important role in the induction of P_*BAD*_ by stimulating the opening of the DNA loop and either the binding of the RNA polymerase or the transition to the open complex (Schleif 2010). In the genome of *G. oxydans*, there is only a single protein (GOX0974/GOX_RS06010) predicted to belong to the CRP/FNR superfamily of transcriptional regulators (Korner et al. 2003; Kranz et al. 2017; Prust et al. 2005). According to our data, this protein is a member of the FNR family and not a cAMP-binding protein of the CRP family (unpublished). Therefore, CRP appears to be absent in *G. oxydans*. Furthermore, with plasmid pBBR1MCS-5-P_*BAD*_*-mNG* missing the *araC* gene, we confirmed that the induction by l-arabinose specifically depended on AraC and not on an endogenous *G. oxydans* protein. The absence of AraC and therefore the missing repression of P_*BAD*_ was not sufficient to allow transcription from P_*BAD*_ in *G. oxydans*. A CRP likely absent in *G. oxydans* could be the reason. This is important in view of leakiness, yet we cannot rule out that in the absence of AraC P_*BAD*_ is somehow repressed by a *G. oxydans* protein that is displaced from P_*BAD*_ when AraC is present. Nevertheless, the activation indicated that in *G. oxydans*, AraC was functional and required as a transcriptional activator of P_*BAD*_, thus heterologous AraC bound to the respective promoter DNA in the cytoplasm and interacted with the RNA polymerase of *G. oxydans*, yet AraC was not required to repress P_*BAD*_. Therefore, using transcriptional activation instead of derepression and considering CRP may circumvent the high leakiness of the classical repressor-based expression plasmids in AAB. However, the reason why AraC-P_*BAD*_ from *E. coli* did not perform well in *G. xylinus* 700178, *G. hansenii* 53582, or *K. rhaeticus* iGEM remains unclear (Teh et al. 2019). We used the *araC*-P_*BAD*_ sequence from *E. coli* K12 MC4100 that exhibits much better codon usage frequencies in *G. oxydans* for five rare *E. coli* K12 MG1655 codons located in the C-terminal helix-turn-helix region of AraC responsible for DNA binding. The effects of the different *araC* codon usage frequencies from MC4100 vs. MG1655 in AAB are unknown. Furthermore, differences in the constructed plasmids such as terminators used and the orientations of plasmid-based reporter and resistance genes to each other, as well as further little sequence-specific differences may affect the formation or stability of their transcripts and the resulting translation, thereby affecting the overall performance of the AraC-P_*BAD*_ systems in AAB. This can already be seen in the result that even in *G. oxydans* with plasmid pBBR1MCS-5-*araC*-P_*BAD*_-MCS-*mNG*, which was very similar to the extremely well-performing plasmid pBBR1MCS-5-*araC*-P_*BAD*_-*mNG*, only approximately 40% of the maximal specific mNG fluorescence was found. This indicated that already the single G to C exchange between the RBS and the ATG start codon to create an *Nde*I site or the short remaining MCS sequence from the *Xho*I site (CTCGAG) after the stop codon directly upstream of the terminator sequence and overlapping with it by the last G, or even both, somehow negatively affected the resulting *mNG* transcript or its translation more drastically than one would expect. Further studies are required to analyze these sequence effects in AAB in more detail.

For induction of the AraC-P_*BAD*_ system in *E. coli*, typically a concentration in the range from 0.001 to 0.2% of l-arabinose is sufficient (Guzman et al. 1995; Narayanan et al. 2006). In this study with *G. oxydans* and plasmid pBBR1MCS-5-*araC*-P_*BAD*_*-mNG*, a range of approximately 0.1 to 1% of l-arabinose was required for minimal and maximal induction. This range is much higher compared with *E. coli* and reflects an altered inducer responsiveness of the AraC-P_*BAD*_ system in *G. oxydans*. The GC-TOF-MS analysis demonstrated that l-arabinose was oxidized by the membrane-bound glucose DH in strain 621H. Nevertheless, in the multi-deletion strain BP.6 almost completely unable to oxidize l-arabinose, the l-arabinose responsiveness of the AraC-P_*BAD*_ system was similar as that of strain 621H. Thus, oxidation and the resulting decrease of l-arabinose did not reduce the responsiveness of the AraC-P_*BAD*_ system, although the oxidation product l-arabinonic acid did not act as an inducer on AraC. Therefore, usage of the AraC-P_*BAD*_ system in *G. oxydans* is not limited to strains that are unable to oxidize l-arabinose.

The lowered responsiveness of the AraC-P_*BAD*_ system in *G. oxydans* to l-arabinose might be caused by a very low uptake of the sugar into the cell. Attempts to improve uptake by expression of the *E. coli araE* gene encoding a secondary transporter for l-arabinose did not increase the responsiveness and even had a negative effect on the expression of the reporter gene *mNG* in our case. The reason for this result is unclear. It might be related to the co-expression of *araE* with *araC* resulting in a longer transcript than *araE* or *araC* mRNA alone. The longer transcript could be less stable or could form secondary structures that negatively affect the translation process, both yielding less AraC activator protein. Additional experiments with separate expression of *araE* from a constitutive promotor independent of *araC*-P_*BAD*_ or alternative arabinose transporter genes are necessary to elucidate the potential to improve the responsiveness of the *araC*-P_*BAD*_ system in *G. oxydans* (Khlebnikov et al. 2001). An endogenous l-arabinose transporter is not known in *G. oxydans* (Prust et al. 2005). A BLAST search with the *E. coli* AraE protein sequence in the *G. oxydans* proteome revealed three proteins with 46 to 56% sequence identity. GOX0808 (56% identity) is annotated as a galactose-proton symporter, GOX0649 (51% identity) as a sugar-proton symporter, and GOX1971 (46% identity) also as a galactose-proton symporter. One or several of these transporters might be promiscuous and poorly take up l-arabinose as a side reaction. No data are available on the substrates, properties, and physiological functions of these transporters.

With respect to the question if l-arabinose can be degraded within *G. oxydans* cells, our results in which this sugar was used in complex medium as sole supplement and as co-substrate with d-mannitol argue against this possibility. With l-arabinose alone strain 621H showed maximally one doubling while strain BP.6 showed no growth at all. This suggested that oxidation of l-arabinose somewhat contributed to generate energy in 621H for growth, yet not in BP.6 unable to oxidize l-arabinose. In *E. coli* and other bacteria, l-arabinose is catabolized *via* an initial isomerization to l-ribulose catalyzed by AraA, followed by phosphorylation to l-ribulose 5-phosphate catalyzed by AraB, and epimerization to d-xylulose 5-phosphate catalyzed by AraD (Englesberg 1961; Englesberg et al. 1962). In *G. oxydans*, homologs of AraA and AraD are absent and only a protein annotated as ribulokinase (GOX2186) showing 26% sequence identity to *E. coli* AraB was found. Therefore, l-arabinose taken up by *G. oxydans* is probably not converted to an intermediate like d-xylulose 5-phosphate that could be catabolized in the pentose phosphate pathway.

The oxidation of l-arabinose by *G. oxydans* 621H did not affect the inducibility of P_*BAD*_, yet as a side effect the growth medium was much more acidified due to the formation of l-arabinonic acid. Therefore, when expressing target genes encoding pH-sensitive proteins in batch cultures without pH control, the use of a *G. oxydans* strain like BP.6 that lacks the ability to oxidize l-arabinose is beneficial to preserve the activity of the target protein in the stationary phase. In the case of strain 621H, additional acidification of the medium due to l-arabinonic acid formation resulted in a severe loss of UidA activity and mNG fluorescence in the stationary phase. Both, UidA activity and mNG fluorescence could be partly or almost fully recovered by solely transferring the cells into fresh medium adjusted to pH 6, indicating that at least the mNG protein was hardly degraded. The activity of the β-d-glucuronidase UidA is reduced by half when the pH is lowered to 4.3 compared with the activity in the optimal range of pH 5.0 to 7.5 (Jefferson et al. 1986). For mNG, it was shown that between pH 5 and 6, three different protonated forms of the chromophore are present. When lowering the pH from 6 to 4, the acidic form becomes predominant, which decreases the fluorescence intensity by half and at pH 3 the mNG fluorescence is completely lost (Steiert et al. 2018). Our flow cytometry analysis revealed the occurrence of two subpopulations of 621H cells with reduced mNG fluorescence 26 h after induction in medium with a pH of 3.3. These two subpopulations might reflect a different progress in cytoplasmic acidification of 621H cells coping with pH 3.3 in the stationary phase. After resuspension in fresh medium with pH 6, mNG fluorescence was recovered and the two subpopulations merged into a single population. In strain BP.6, which is unable to oxidize l-arabinose, flow cytometry revealed that the acidification of the medium from pH 6 to 4.4 had only a marginal effect on mNG fluorescence. As the studies with isolated mNG demonstrated a strong decrease in fluorescence at pH 4.4 (Steiert et al. 2018), this result indicates that the intracellular pH in BP.6 cells must be higher than that in 621H cells. Bacteria have evolved various mechanisms for pH homeostasis in order to maintain the cytoplasmic pH in a range that enables survival and growth (reviewed in, for example, Baker-Austin and Dopson 2007; Follmann et al. 2009; Krulwich et al. 2011). For *G. oxydans*, the mechanisms of pH homeostasis have not yet been studied. We previously compared global gene expression of *G. oxydans* 621H cells grown at pH 4 and at pH 6 and observed 35 genes which were upregulated at pH 4 more than twofold and 37 genes that were downregulated more than twofold (Hanke et al. 2012). Obvious mechanisms for pH homeostasis could not be deduced from these data. In summary, the comparison of mNG fluorescence in strains 621H and BP.6 suggested that *G. oxydans* is well able to perform reasonable pH homeostasis at pH 4.4 and less able at pH 3.3. However, when maintaining the medium at pH 6, *e*.*g*., by cultivation of the cells with pH control, the acidification due to l-arabinose oxidation can be avoided also in *G. oxydans* 621H, preserving the activity of proteins synthesized under the control of P_*BAD*_. Together, our results demonstrate the functionality and potential of l-arabinose-inducible gene expression as a tool for *G. oxydans* and possibly also for other AAB.

## Electronic supplementary material

ESM 1 (PDF 1210 kb).
