# Supplementary material for: A tunable l-arabinose-inducible expression plasmid for the acetic acid bacterium Gluconobacter oxydans
Source: Appl Microbiol Biotechnol. 2020 Sep 25;104(21):9267–82. doi: 10.1007/s00253-020-10905-4 (PMC7567684; doi:10.1007/s00253-020-10905-4)
Supplement: Supplementary file 1 — (PDF 1210 kb). [file 253_2020_10905_MOESM1_ESM.pdf]

Journal: Applied Microbiology and Biotechnology

A tunable L-arabinose-inducible expression plasmid for the acetic acid bacterium *Gluconobacter oxydans*

Philipp Moritz Fricke, Tobias Link, Jochem Gätgens, Christiane Sonntag, Maike Otto, Michael Bott and Tino Polen*

Forschungszentrum Jülich GmbH, IBG-1: Biotechnology, Institute of Bio- and Geosciences
52425 Jülich, Germany

* for correspondence:	Dr. Tino Polen	
	e-mail:	t.polen@fz-juelich.de
	phone:	+49 (0)2461 61 6205
	fax:	+49 (0)2461 61 2710

Supplementary Data

Table S1	List of DNA oligonucleotides
Table S2	mRNA level changes 30 min after a pulse of 1% (w/v) L-arabinose
Table S3	Selected codon usage frequencies of *E. coli araC* in *G. oxydans* 621H
Fig. S1	GC-TOF MS analysis of L-arabinose oxidation by *G. oxydans* strains
Fig. S2	Schemes of the pBBR1MCS-5-based reporter plasmids from Gibson cloning
Fig. S3	Test of AraC-specific induction of P_{BAD} in strain 621H
Fig. S4	Test of L-arabinonic acid as inducer
Fig. S5	Influence of the L-arabinose transporter AraE on the inducibility of P_{BAD}
Fig. S6	Gain of reporter protein activity in strain 621H by pH-adjusted medium
Fig. S7	Functionality of the multiple cloning vector pBBR1MCS-5-*araC*-P_{BAD}-MCS

Table S1 DNA oligonucleotides used in this study. Sequences in bold represent extensions for the terminator sequences T7 or BBa_B1002 of the iGEM parts library. Sequences underlined represent the Shine-Dalgarno sequence used in *G. oxydans*. Gray-boxed sequences represent *Nde*I and *Xho*I restriction sites.

Name	DNA sequence 5'-> 3'
PF1	AACAAAAGCTGGGTACCAATTATGACAACTTGACGGCTAC
PF2	GTAACATATCATATCTCCTTGGACTGTTAGCCC
PF3	GCTAACAGTCCAAGGAGATATGATATGTTACGTCCTGTAG
PF4	GGGCGAATTGGAGCTCGCGAAAAAACCCCGCCGAAGCGGGGTTTTTTGCGTCATTGTTTGCC
PF5	TCCTTGGACTGTTAGCCCAAAAAACGGGTA
PF6	TTTTTGGGCTAACAGTCCAAGGAGATATGATATGG
PF7	GCGAATTGGAGCTCGCGAAAAAACCCCGCCGAAGCGGGGTTTTTTGCGTTACTTATACAGCTCATCCATG
PF8	CAATTGGGGCGCGCCCTGCAGGTCTAGAACTAGTGGATCC**AAACCAATTGTCCATATTGCATCAGAC**
PF9	CTAAAGGGAACAAAAGCTGGGTACCGGGCCCCCCCTCGAGCATAGGGCGAATTGGAGC
PF10	CAATTATTACCTCCACGGGGAGAGCCTGAGCAAACTGGCCTCAGG**TACCATAACCCCTTGGGGCCTCTAAACGGGTCTTGAGGGGTTTTTTG**GCTTCAGACGCCGATATTTCTCA
PF11	GTCATAATTGGTACCCAGCTTTTGTTCCCTTTAGTGAGGGCGCGCCTCCAAGGAGATATGATATGGTTACTATCAATACGG
PF12	ACGGTCACACTGCTTCCGGTAGTCAATAAACCGGTGGCGCGCCCTCA
PF13	CGGCCGCCACCGCGGTGGAGCTCCATATGATATCTCCTTGGACTGTTAGC
PF14	GCTTATCGATACCGTCGACCTCGAG**CGCAAAAAACCCCGCTTCGGCGGGGTTTTTTCGC**GGTACCCAGCTTTTGTTCC
PF15	CTTAATGAATTACAACAGTTTTTATGCATGCGCCCAATACGCA
PF16	ACCTTTAGACACCATATGATATCTCCTTGG
PF17	CCAAGGAGATATCATATGGTGTCTAAAGGT
PF18	TAAAGGGAACAAAAGCTGGGTAC**CGCGAAAAAACCCCGCCGAAGCGGGGTTTTTTGCG**CTCGAGTTACTTATACAGCTCATCCATG

Table S2 The top 10 genes showing increased or decreased mRNA ratios in *G. oxydans* 621H 30 min after a pulse of 1% (w/v) L-arabinose or water (as control) added to shaking flask batch cultures.

Locus Tag	Annotation	L-Ara/ H_2O
GOX0707	DNA starvation/stationary phase protection protein Dps	1.97
GOX1952	hypothetical protein GOX1952	1.90
GOX0726	hypothetical protein GOX0726	1.84
GOX2257	hypothetical protein GOX2257	1.81
GOX1007	hypothetical protein GOX1007	1.79
GOX1534	hypothetical protein GOX1534	1.70
GOX0943	hypothetical protein GOX0943	1.61
GOX1799	protein translocase subunit YajC	1.59
GOX1620	two component response regulator	1.56
GOX0809	L-asparaginase II	1.54
GOX0369	50S ribosomal protein L24	0.60
GOX0945	TonB-dependent outer membrane receptor	0.60
GOX0310	NAD(P) transhydrogenase subunit alpha	0.60
GOX0531	ExbD protein	0.59
GOX2590	pGox1, hypothetical protein GOX2590	0.59
GOX1857	PQQ-containing dehydrogenase 1, inositol dehydrogenase	0.59
GOX0532	ExbB protein	0.57
GOX0422	hypothetical protein GOX0422	0.56
GOX0758	porin	0.56
GOX0536	hydroxamate-type ferrisiderophore receptor	0.46

Table S3 Codon usage frequencies (%) in *G. oxydans* 621H of the nine codons of *araC* that are different in the *E. coli* K12 derivatives MG1655 and MC4100. Codons with strongly increased frequencies when using *araC* from MC4100 instead of MG1655 are marked in bold.

AraC position	Amino acid	MG1655 codon	% in *G. oxydans*	MC4100 codon	% in *G. oxydans*
16	N	AAC	16.9	AAU	10.3
65	V	GUC	30.7	GUU	12.5
118	G	GGU	16.1	GGG	16.0
231	R	CGC	30.0	CGU	16.1
232	I	AUU	11.7	AUC	**36.1**
233	S	AGU	4.5	AGC	**15.2**
241	T	ACU	3.2	ACC	**21.0**
263	R	CGA	3.2	CGG	**17.9**
276	F	UUU	9.5	UUC	**25.3**

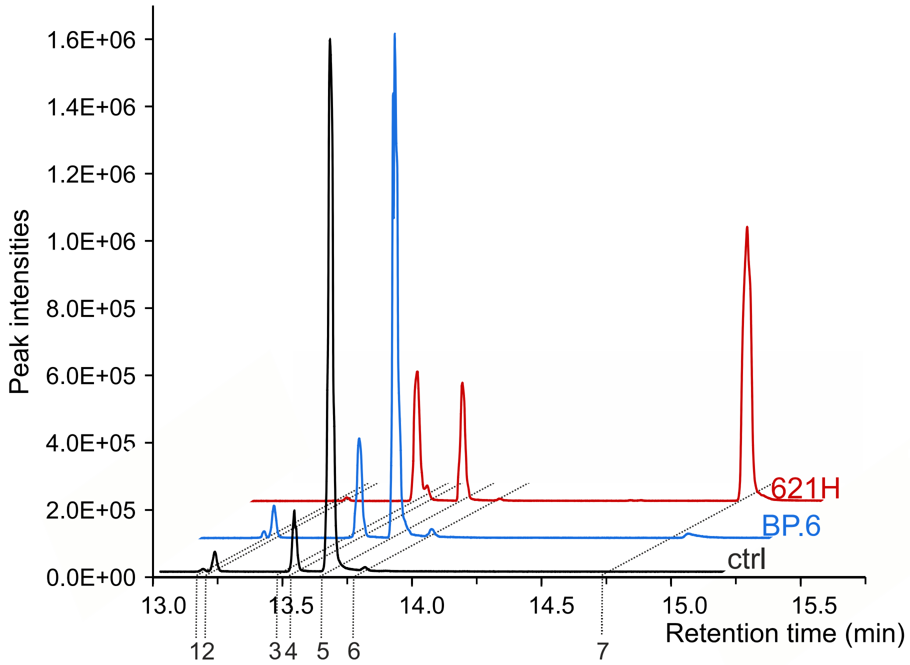

1 Arabinofuranose (4 TMS) (1), RI 1595, 13.79 min

2 Arabinopyranose (4 TMS), RI 1599, 13.21 min

3 Arabinonic acid-1,4-lactone (3 TMS), RI 1629, 13.49 min

4 Arabinopyranose (4 TMS), RI 1631, 13.52 min

5 Arabinose (MeOx 4 TMS), RI 1646, 13.65 min

6 Arabinofuranose (4 TMS) (2), RI 1660, 13.79 min

7 Arabinonic acid (5 TMS), RI 1766, 14.74 min

Fig. S1 Chromatograms of GC-TOF-MS analysis of L-arabinose oxidation by *G. oxydans* strains 621H and BP.6. Cell suspensions with an OD_{600} of 1.6 were incubated for 24 h (t24) at 30°C and 180 rpm in biotransformation buffer supplemented with 1% (w/v) L-arabinose. Then cell-free culture supernatant was prepared for GC-TOF-MS analysis. Peak 6 at retention time 14.74 min corresponds to arabinonic acid according to mass spectrometric identification and was absent in the cell-free control sample (ctrl) and in the t0 samples of all strains (not shown).

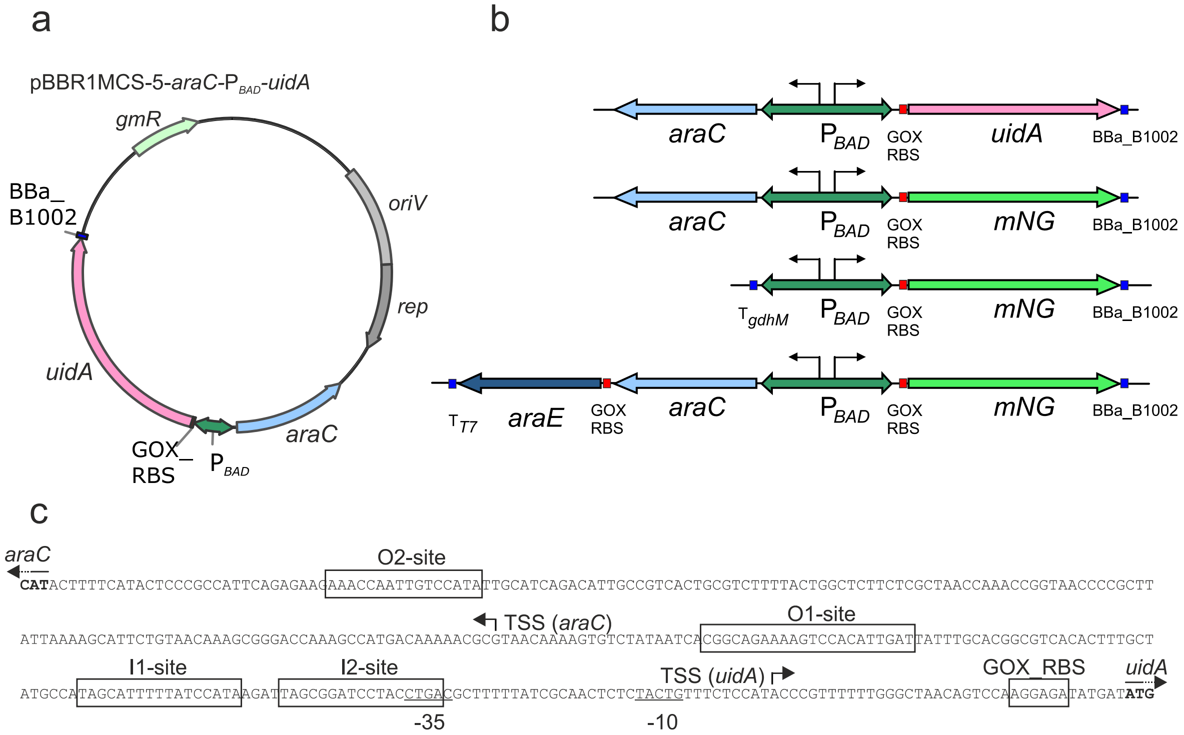

Fig. S2 Schemes of the pBBR1MCS-5-based reporter plasmids with gentamicin resistance cassette (*gmR*) and sequence information. a) Organization of plasmid pBBR1MCS-5-*araC*-P_{BAD}-*uidA* carrying β-D-glucuronidase enzyme reporter gene *uidA* with terminator BBa_B1002 expressed from P_{BAD} with adjacent *araC* gene. b) Variants of plasmid inserts with reporter gene *uidA* or *mNG* to test the L-arabinose-induced AraC-dependent expression and the effect of the L-arabinose transporter AraE. c) Sequence details of the P_{BAD} region with AraC binding sites. The *G. oxydans* ribosomal binding site (GOX RBS) sequence and distance to the translational start of the reporter genes is exemplarily shown for *uidA*. T_{gdhM}: terminator sequence of *gdhM* (GOX0265); T_{T7}: T7 terminator sequence

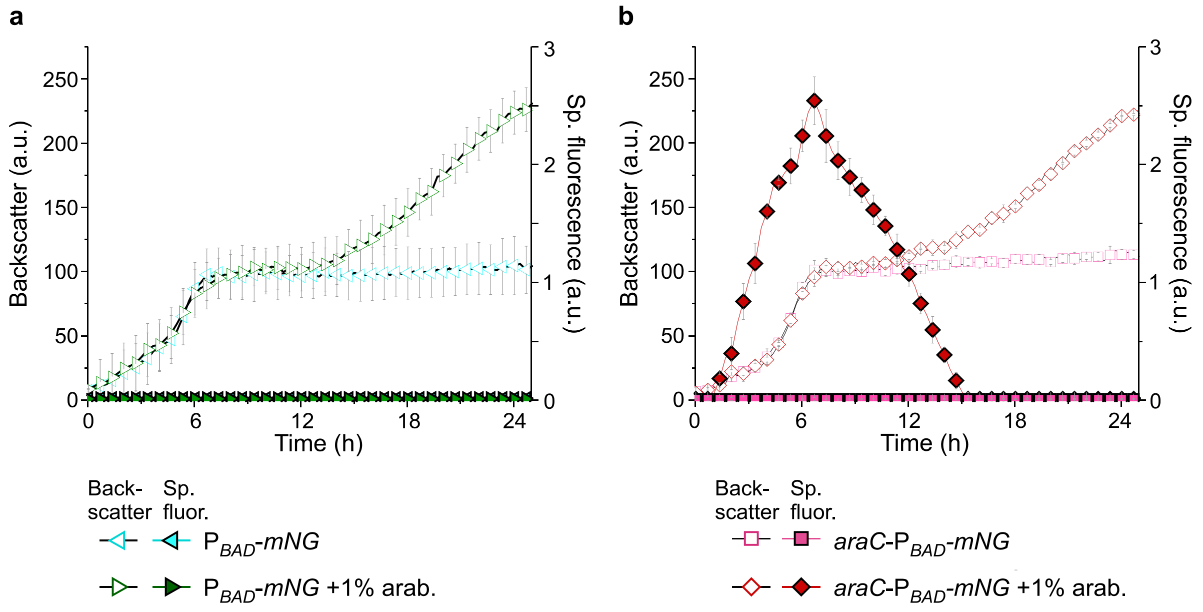

Fig. S3 Inducibility of P$_{BAD}$ in the absence (a) and presence (b) of the regulator AraC. *G. oxydans* 621H carrying pBBR1MCS-5-P$_{BAD}$-*mNG* (a) or pBBR1MCS-5-*araC*-P$_{BAD}$-*mNG* (b) were grown in complex D-mannitol medium with and without 1% (w/v) L-arabinose.

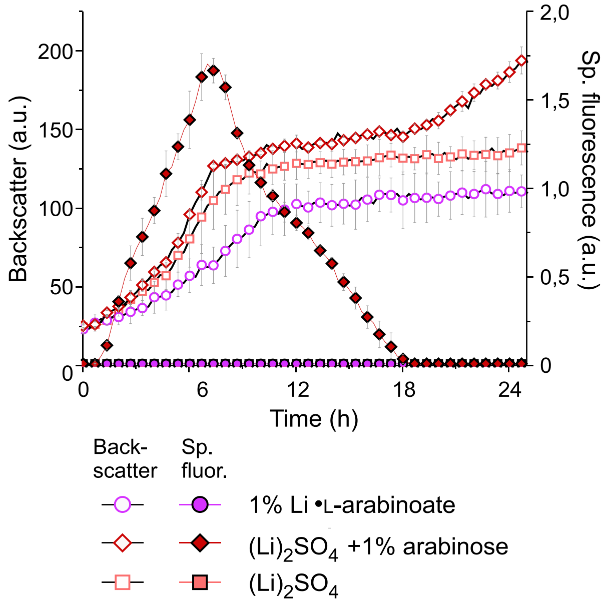

Fig. S4 BioLector cultivations to analyze P*BAD* induction by L-arabinonic acid. *G. oxydans* 621H carrying pBBR1MCS-5-*araC*-P*BAD*-*mNG* was grown in D-mannitol medium without or with 1% (w/v) L-arabinonic acid (the pH of the medium was adjusted to pH 6 after adding the Li^+ salt stock solution). As a control, non-induced and L-arabinose-induced cells were supplemented with the same concentration of Li^+ as $(Li)_2SO_4$. Expression of *mNG* is indicated as the specific fluorescence calculated as the ratio of the absolute mNG fluorescence and the backscatter. Data represent mean values ± SD from three biological replicates.

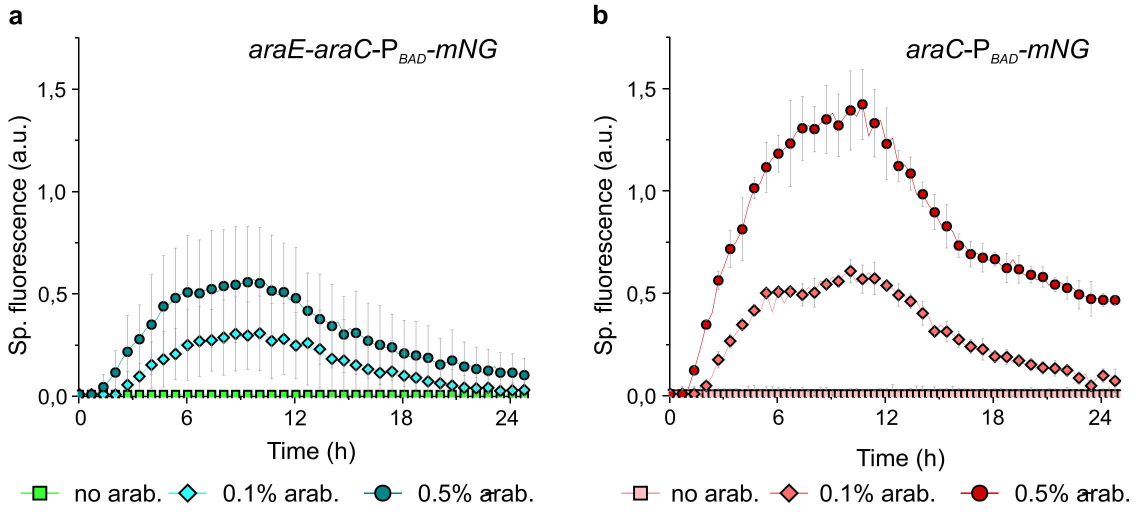

Fig. S5 Influence of the L-arabinose transporter AraE on the inducibility of P$_{BAD}$ measured as specific mNG fluorescence. *G. oxydans* 621H carrying pBBR1MCS-5-*araE-araC*-P$_{BAD}$-*mNG* (a) or pBBR1MCS-5-*araC*-P$_{BAD}$-*mNG* (b) were grown in D-mannitol medium with and without 0.1% or 0.5% (w/v) L-arabinose.

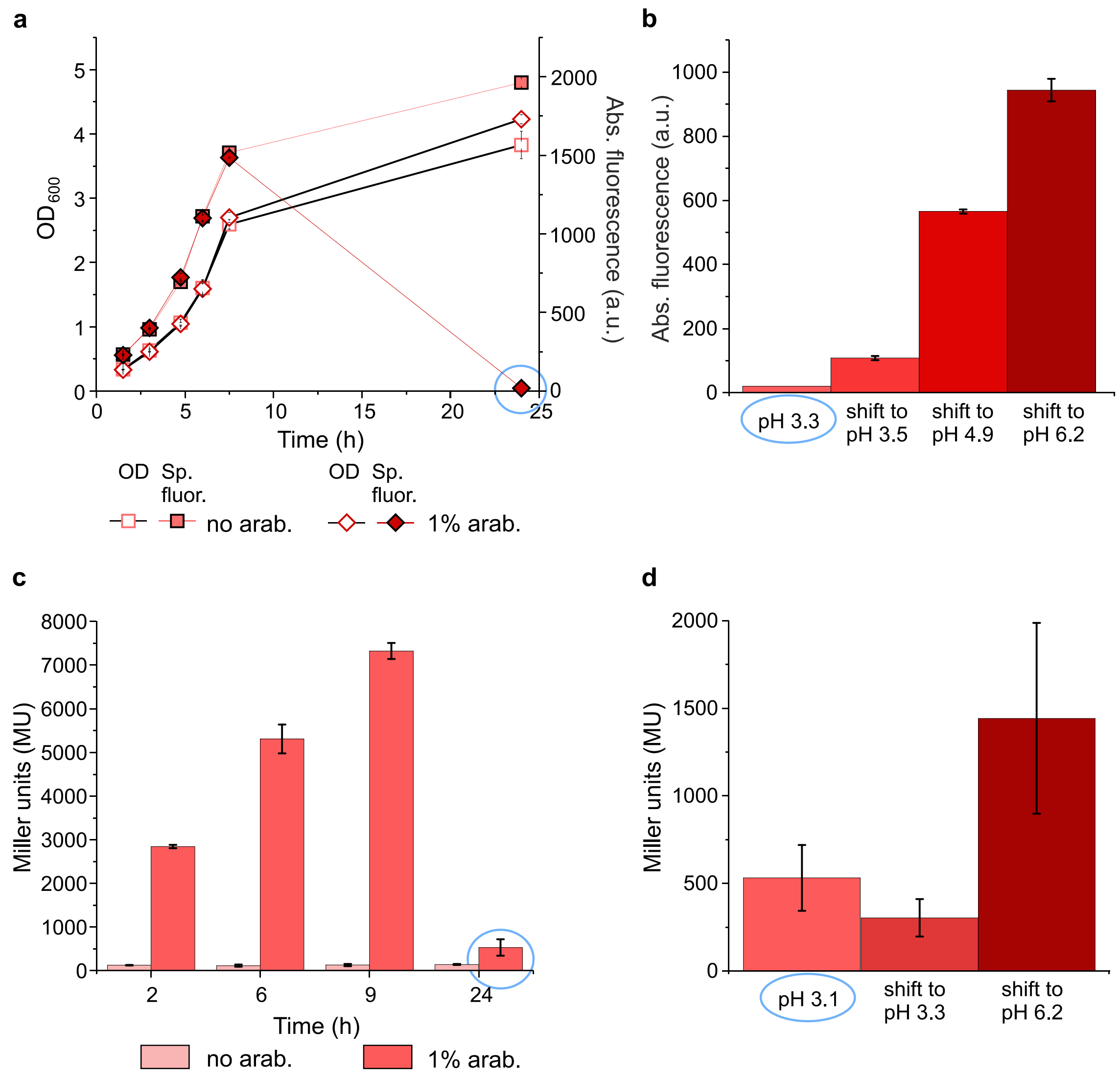

Fig. S6 Dependence of mNG fluorescence and UidA activity in *G. oxydans* cells on pH.
a) Growth of strain 621H (OD_{600}, open symbols) carrying pBBR1MCS-2-$P_{GOX0264}$-*mNG* grown in the absence and presence of 1% (w/v) L-arabinose. The mNG fluorescence (closed symbols) was measured in a Tecan reader. In the culture supplemented with 1% (w/v) L-arabinose the pH of the growth medium was 3.3 at the end of the cultivation (24 h) where the mNG signal was drastically decreased.
b) The decreased mNG fluorescence in 621H cells at 24 h (a) was gradually increased after transferring the cells into fresh L-arabinose- and D-mannitol-free medium adjusted to pH 3.5, 4.9 or 6.2. After 1 h of incubation the mNG fluorescence was determined in a Tecan reader.
c) UidA activities (MU) in strain 621H carrying the plasmid pBBR1MCS-5-*araC*-P_{BAD}-*uidA* during growth with and without L-arabinose. In the culture supplemented with 1% (w/v) L-arabinose the pH of the growth medium was 3.1 at the end of the cultivation (24 h) where the UidA activity was drastically decreased.
d) The decreased UidA activity in 621H cells at 24 h (c) was approximately 3-fold increased by transferring cells into fresh L-arabinose- and D-mannitol-free medium adjusted to pH 6.2 after 1 h of incubation. The UidA activities were determined in Miller assays. Data represent mean ± SD from three biological replicates.

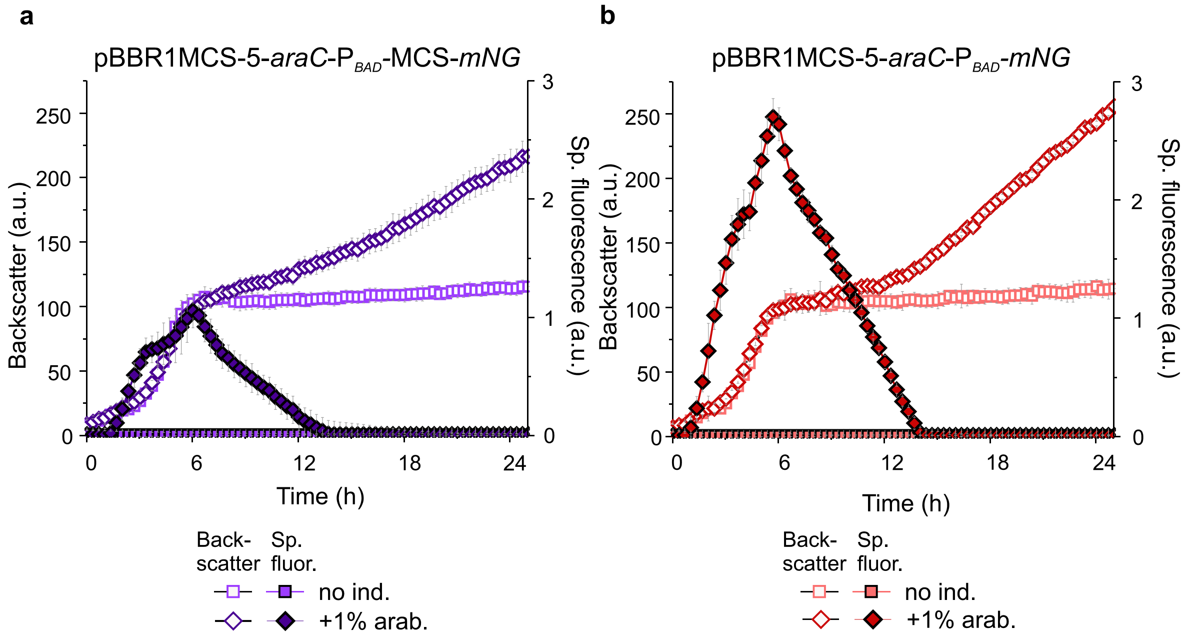

Fig. S7 L-arabinose-induced *mNG* reporter expression based on the multiple cloning vector pBBR1MCS-5-*araC*-P_{BAD}-MCS (a) in comparison with the pBBR1MCS-5-*araC*-P_{BAD}-*mNG* test plasmid (b). The plasmids were compared in strain 621H grown in the absence and presence of 1% (w/v) L-arabinose in microscale BioLector cultivations.
